# MicroRNA paraffin-based studies in osteosarcoma reveal reproducible independent prognostic profiles at 14q32

**DOI:** 10.1186/gm406

**Published:** 2013-01-22

**Authors:** Andrew D Kelly, Benjamin Haibe-Kains, Katherine A Janeway, Katherine E Hill, Eleanor Howe, Jeffrey Goldsmith, Kyle Kurek, Antonio R Perez-Atayde, Nancy Francoeur, Jian-Bing Fan, Craig April, Hal Schneider, Mark C Gebhardt, Aedin Culhane, John Quackenbush, Dimitrios Spentzos

**Affiliations:** 1 Division of Hematology/Oncology, Sarcoma Program, Department of Medicine, Beth Israel Deaconess Medical Center, Harvard Medical School, 330 Brookline Avenue, Boston, MA 02215, USA; 2 Center for Cancer Computational Biology, Department of Biostatistics and Computational Biology, Dana Farber Cancer Institute, Boston, MA 02215, USA; 3 Bioinformatics and Computational Genomics, Institut de Recherches Cliniques de Montréal, 110 Avenue Des Pins O, Montreal, Quebec H2W 1R7, Canada; 4 Division of Hematology/Oncology, Department of Medicine, Boston Children's Hospital, Harvard Medical School, 300 Longwood Avenue, Boston, MA 02215, USA; 5 Department of Pathology, Beth Israel Deaconess Medical Center, Harvard Medical School, 330 Brookline Avenue, Boston, MA 02215, USA; 6 Department of Pathology, Boston Children's Hospital, Harvard Medical School, 300 Longwood Avenue, Boston, MA 02215, USA; 7 Illumina Inc., 5200 Illumina Way, San Diego, CA 92122, USA; 8 Molecular Genetics Core Facility, Boston Children's Hospital, Harvard Medical School, 300 Longwood Avenue, Boston, MA 02215, USA; 9 Department of Orthopedic Surgery, Beth Israel Deaconess Medical Center, Harvard Medical School, 330 Brookline Avenue, Boston, MA 02215, USA

## Abstract

**Background:**

Although microRNAs (miRNAs) are implicated in osteosarcoma biology and chemoresponse, miRNA prognostic models are still needed, particularly because prognosis is imperfectly correlated with chemoresponse. Formalin-fixed, paraffin-embedded tissue is a necessary resource for biomarker studies in this malignancy with limited frozen tissue availability.

**Methods:**

We performed miRNA and mRNA microarray formalin-fixed, paraffin-embedded assays in 65 osteosarcoma biopsy and 26 paired post-chemotherapy resection specimens and used the only publicly available miRNA dataset, generated independently by another group, to externally validate our strongest findings (*n *= 29). We used supervised principal components analysis and logistic regression for survival and chemoresponse, and miRNA activity and target gene set analysis to study miRNA regulatory activity.

**Results:**

Several miRNA-based models with as few as five miRNAs were prognostic independently of pathologically assessed chemoresponse (median recurrence-free survival: 59 months versus not-yet-reached; adjusted hazards ratio = 2.90; *P *= 0.036). The independent dataset supported the reproducibility of recurrence and survival findings. The prognostic value of the profile was independent of confounding by known prognostic variables, including chemoresponse, tumor location and metastasis at diagnosis. Model performance improved when chemoresponse was added as a covariate (median recurrence-free survival: 59 months versus not-yet-reached; hazard ratio = 3.91; *P *= 0.002). Most prognostic miRNAs were located at 14q32 - a locus already linked to osteosarcoma - and their gene targets display deregulation patterns associated with outcome. We also identified miRNA profiles predictive of chemoresponse (75% to 80% accuracy), which did not overlap with prognostic profiles.

**Conclusions:**

Formalin-fixed, paraffin-embedded tissue-derived miRNA patterns are a powerful prognostic tool for risk-stratified osteosarcoma management strategies. Combined miRNA and mRNA analysis supports a possible role of the 14q32 locus in osteosarcoma progression and outcome. Our study creates a paradigm for formalin-fixed, paraffin-embedded-based miRNA biomarker studies in cancer.

## Background

Osteosarcoma is the most common primary bone malignancy, disproportionally affecting children and young adults [1]. Overall five-year survival rates for newly diagnosed osteosarcomas range from 40% to 75% [2]. Standard treatment consists of two to three rounds of chemotherapy, followed by definitive resection, and additional adjuvant chemotherapy. Although pathologically assessed chemoresponse is a useful surrogate for long-term outcome, it is not always tightly correlated with recurrence patterns and survival. Patients whose tumors show high levels of necrosis following preoperative chemotherapy have a uniformly good prognosis (up to 90% cure rates) whereas those with lower levels of necrosis have variable outcomes, potentially including long-term remissions. Despite work on genetic characterizations of this disease, there are currently no good biomarkers for osteosarcoma outcome following standard treatment [3,4]. This has prevented effective recurrence risk stratification and may explain treatment strategies for osteosarcoma remaining unchanged for almost 20 years. Previous studies have reported gene expression profiles associated with chemoresponse in human cohorts as well as genes associated with survival in a dog osteosarcoma model [5-7], thereby providing important biological insights, but sample limitations did not allow development of a clinical prognostic signature for recurrence and survival, which remains an unmet need.

MicroRNAs (miRNAs) are critical regulators of cancer biology with a probable role in different sarcomas [8]. Osteosarcoma-focused studies found differentially expressed miRNAs between osteosarcoma tissue and normal osteoblasts, and implicated miRNAs in chemoresistance [9-12] or *in vitro *proliferation and metastasis [13,14]. A recent study reported miRNAs predictive of chemoresponse and miRNAs associated with a binary metastasis endpoint in a human cohort, and provided biological context for their role [7]. However, to date, human outcome studies have been limited by small patient sample sizes (n < 30, a common limitation when studying rare diseases). Thus, a formal gene or miRNA model predictive of outcomes using human osteosarcoma clinical specimens has not yet been developed. This effort is further limited by the rarity of frozen tissue resources, with long-term outcome annotation suggesting that formalin-fixed, paraffin-embedded (FFPE) tissue may be a critical alternative resource for such studies.

In the largest osteosarcoma profiling study to date, we developed miRNA models with independent predictive value for recurrence and overall survival (OS) from FFPE human osteosarcoma diagnostic biopsy specimens. Prognostic miRNAs were mainly clustered on a chromosomal locus recently reported to be linked to osteosarcoma [10,15]. We utilized the only other publicly available osteosarcoma miRNA dataset that included outcome annotations and were able to independently validate the prognostic value of many of our candidate miRNAs. Lastly, we performed complementary assessment of chemoresponse using both static and dynamic paired expression patterns. Our study sets a paradigm for profiling studies using FFPE samples in rare tumors.

## Methods

### Paraffin-based human osteosarcoma cohort

We used 91 FFPE osteosarcoma samples from the pathology archives of Beth Israel Deaconess Medical Center and Boston Children's Hospital. The cohort included 65 diagnostic biopsy specimens and 26 paired surgical resection specimens (Table 1 and Table S1 in Additional file 1). A protocol for archival tissue collection was approved by Institutional Review Board at both institutions with a waiver of consent.

**Table 1:** Clinical characteristics of the osteosarcoma cohort

Characteristic	Number (*n *= 65)
Age, years^a, b^	
Median	12
Range	3 to 76
Gender	
Male	30 (46%)
Female	35 (54%)
Specimens	
Biopsy	65
Resection	26
Chemosensitive tumors	32 (49%)
Tumor location	
Axial	3
Appendicular	62
Events	
Recurrence	23
Death	14
Metastases at diagnosis	
No	54
Yes	11
Preoperative chemotherapy	
MAP only	42
Other than MAP (for example MAPIE, IE)	23

**^a^**Two patients were over 35 years old; ^b^one had prior Paget's bone disease.

### Formalin-fixed, paraffin-embedded RNA isolation, whole genome and miRNA profiling, quality control and processing

FFPE samples were cut into 10 μm sections. Total RNA was isolated using the Qiagen RNeasy FFPE protocol (**Qiagen, Valencia, CA, USA**). miRNA expression profiling was performed for all 91 FFPE samples using miRNA cDNA-mediated annealing, selection, extension and ligation (DASL) assays (Illumina, Hayward, CA, USA), containing probes for 1,146 miRNAs [16,17]. Whole genome DASL arrays, containing probes for 29,285 transcripts, were used for profiling all 26 surgical resection specimens in addition to 43 of the biopsy specimens and were conducted as previously described [18-20]. Assays were run at the Molecular Genetics Core Facility at Boston's Children's Hospital. The DASL assay is a bead-based method for expression profiling of degraded RNA, such as that extracted from FFPE samples [16-24]. Raw miRNA and mRNA DASL data have been deposited in the National Center for Biotechnology Information's Gene Expression Omnibus [GSE:39058] [25]. Dataset quality control metrics included the number of probes significantly detected (*P *< 0.01), average signal intensity, 95^th ^percentile signal intensity and housekeeping gene signal intensity (Additional file 2). All 91 miRNA assays passed quality control criteria. There was no correlation between specimen storage age and quality (Additional file 2). Of the mRNA expression profiling assays, 42 passed quality control criteria including 37 biopsy specimens and 5 surgical resection specimens. After excluding failed samples, data were processed by variance stabilizing transformation and quantile normalization using the Lumi package in R [26,27]. To minimize noise from uninformative probes, we filtered out miRNAs with an expression variance across the cohort in the bottom 33%, and we filtered out mRNAs with an expression variance in the bottom 90%.

### Computational survival analysis and miRNA activity methods

Recurrence-free survival (RFS), OS and gene set expression comparison analyses (GSA) were done using the National Cancer Institute Biometric Research Branch ArrayTools software [28,29]. For recurrence and survival analysis, differentially expressed miRNAs and mRNAs were identified using standard statistical methods employed by the software. Risk prediction models were generated using an implementation of the supervised principal components method originally described by Bair and Tibshirani [30]. miRNA regulatory activity analysis was performed using an adoption of the regulatory effects scoring method developed by Cheng *et al*. [31]. mRNA data were imported into the R environment and miRNAs were called as significantly differentially activated if the regulatory effects scoring was associated with a *P*-value of 0.05 (false discovery rate (FDR) < 0.1). The script used in this analysis has been uploaded as Script S1 in Additional file 3. The miRanda-based GSA target gene algorithm was implemented on ArrayTools [28,29,32].

### Ordinal logistic regression modeling and chemotherapy response prediction

To take advantage of the ordinal nature of the chemotherapy response endpoint, we used ordinal logistic regression (OLR) as our primary mathematical modeling tool:

(1) $$\text{ln}\;\theta_{i}=\alpha_{i}-\Sigma_{j}\beta_{j}X_{j}\;\;\theta_{i}=\frac{p\left(Category\leq i\right)}{\left(1-p\left(Category\leq i\right)\right)}$$

(2) $$P\left(Z\in Category\;1\right)=\frac{1}{1+e^{-\left(\alpha_{1}-\sum \beta_{k}X_{k}\right)}}$$

OLR regresses a log-likelihood ratio of one ordered category versus another on continuous independent variables - in this case normalized miRNA or mRNA expression measurements (equation 1) [33] and was implemented using the Design package in the R environment [34,35]. In brief, the sample cohort was randomly split 500 times into training and test sets. After feature selection, an OLR model was trained using each of the 500 training sets, and predicted probabilities were obtained for each respective test set (equation 2). A chemoresponse category was assigned to that with the highest predicted probability. Multiple univariate OLR models were generated using up to 20 miRNAs with the highest individual concordance values and chemotherapy response predictions were assigned based on the geometric means of multiple OLR model-based predictions. We also attempted multivariate prediction using a small number of miRNAs as described in the supplementary methods (Additional file 2). The scripts used to implement OLR have been uploaded as Script S2 in Additional file 4 and Script S3 in Additional file 5.

## Results

### Osteosarcoma miRNA and mRNA assays

We used 65 FFPE primary osteosarcoma diagnostic biopsy specimens from the pathology archives at Beth Israel Deaconess Medical Center and Boston Children's Hospital. We also obtained paired, post-chemotherapy surgical resection specimens for 26 of these patients (Table S1 in Additional file 1). miRNA and whole genome DASL assays were performed as described in the methods section.

### miRNA profiles of recurrence and survival in osteosarcoma

**Figure 1:**
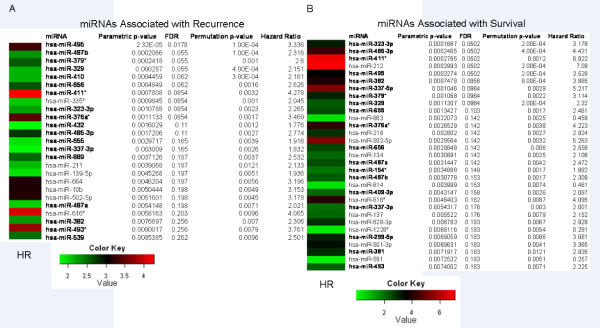
**miRNAs associated with recurrence and survival**. miRNAs significantly associated with **(A) **recurrence and **(B) **survival (*P *< 0.01). Color map displays univariate HRs for recurrence. Bold text denotes miRNAs located at 14q32. FDR, false discovery rate; HR, hazard ratio.

We first sought to identify miRNA and mRNA expression profiles associated with risk for recurrence and OS. Using standard univariate Cox proportional hazards models we identified 25 miRNAs associated with RFS and 31 miRNAs associated with OS (*P *< 0.01; Figure 1A and 1B, respectively). The two sets of miRNAs were highly overlapping and strongly significant when corrected for multiple testing.

**Figure 2:**
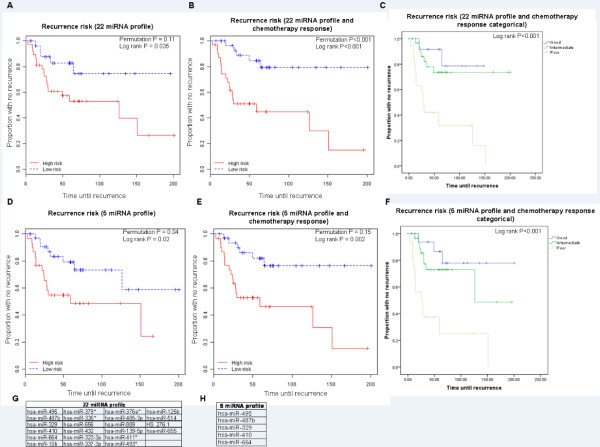
**Recurrence risk prediction**. **(A, D) **Kaplan-Meier analysis of recurrence risk (supervised principal components analysis for the 22 and 5 miRNA profiles). **(B, E) **Kaplan-Meier analysis of recurrence risk for the 22 and 5 miRNA profiles in addition to chemoresponse as a clinical covariate in the model. **(C, F) **Kaplan-Meier analysis of recurrence risk using both 22 and 5 miRNA profiles and chemoresponse as categorical variables (three-group analysis). **(G) **22 miRNA profile. **(H) **5 miRNA profile.

**Figure 3:**
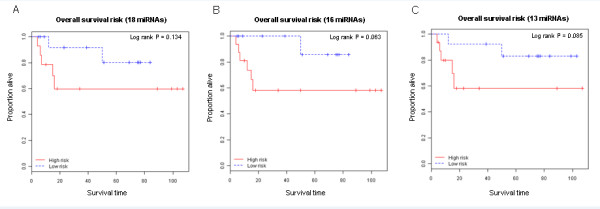
**miRNAs are prognostic of recurrence and survival in an independent external dataset**. Our prognostic miRNAs were used to generate models of OS in an independent external validation dataset. Of the 22 miRNA profile, 18 miRNAs could be mapped on the platform used in the external dataset. **(A-C) **These overlapping miRNAs (as well as smaller subsets of this profile) were used to generate survival risk prediction models. A consistent discriminatory trend was observed in the external dataset, despite a smaller sample size, fewer events and different array platform.

Next, we applied a supervised principal components survival risk prediction method [30] with 10-fold cross-validation and a random permutation test. We found several prognostic models of sizes ranging to at least 25 miRNAs that performed well. Figure 2A and 2D show two indicative examples utilizing two different *P*-value cutoffs for inclusion in the model (*P *< 0.001 and *P *< 0.0075) representing a model with five miRNAs (median RFS: 59 months versus not-yet-reached, hazard ratio (HR) = 2.66, 95% confidence interval (CI): 1.123 to 6.303, log rank *P *= 0.02; permutation *P *= 0.04; Figure 2H), and a model with 22 miRNAs (median RFS: 126 months versus not-yet-reached, HR = 2.77, 95% CI: 1.025 to 7.475, log rank *P *= 0.035; permutation *P *= 0.11; Figure 2G). Because of a limited number of deaths in our study, miRNA-only models were not significantly predictive of OS risk; however, several demonstrated a notable discriminatory trend (Figure 3A).

We repeated the analyses described above using mRNA data, and we identified 66 genes significantly associated with recurrence, and 38 associated with OS (Table S2 in Additional file 6 and Table S3 in Additional file 7; *P *< 0.05). Unlike the miRNA-based analysis, mRNA expression-based models for recurrence and survival did not reach a level of statistical significance likely due to the smaller sample size for this analysis.

### miRNA profiles are prognostic independently of known prognostic factors

We also tested whether miRNA-based risk prediction is independently prognostic of recurrence when controlling for the effect of several known prognostic factors (Table 2). The additional possible confounding covariates we considered were anatomic tumor site, chemotherapy response, presence of metastases at diagnosis, and type of presurgical chemotherapy.

**Table 2:** Multivariate analysis of the miRNA prognostic power adjusting for the effect of known prognostic factors

Univariate hazard ratio (5 miRNA profile)	*P*	Multivariate hazard ratio (5 miRNA profile)		*P*
2.66 (1.12 to 6.30)	0.02	Controlling for chemoresponse	2.67	0.026
		Controlling for metastases at diagnosis	2.40	0.05

Tumor site was not prognostic due to the overwhelming majority of tumors located in the extremities, and preoperative chemotherapy regimen was confounded by the presence of metastasis at diagnosis.

#### Anatomic tumor site

Only three of the patients in the cohort presented with axial tumors whereas the overwhelming majority of tumors were located in the extremities. Thus, the cohort was homogeneous with respect to this covariate, which therefore could not confound the analysis (formal Cox multivariate regression confirmed this as well, *P *= 0.764 with 22 miRNA profile; *P *= 0.666 with 5 miRNA profile).

#### Chemotherapy response

Chemotherapy response, assessed by the degree of tumor necrosis in the primary tumor following preoperative chemotherapy, has been shown to have prognostic value in osteosarcoma. A multivariate Cox model showed that both risk prediction with the 5 miRNA and the 22 miRNA profile and chemotherapy response retained their independent significance (22 miRNA: HR 2.90, *P *= 0.036; chemotherapy response: HR 3.82, *P *= 0.005 and 5 miRNA: HR 2.67, *P *= 0.026, chemotherapy response: HR 3.70, *P *= 0.006).

#### Metastases present at diagnosis

We used metastasis at diagnosis alone to perform multivariate analysis. Formal multivariate Cox regression proved that the miRNA prognostic profile retained its independent prognostic significance when one controls for the effect of metastatic disease at diagnosis (Table 2). As expected, presence of metastasis at diagnosis was a powerful predictor of outcome. However, multivariate Cox model also showed that risk prediction based on the 22 miRNA and 5 miRNA prognostic profiles retained independent prognostic significance for recurrence (HR = 2.27, *P *= 0.115 and HR = 2.40, *P *= 0.050, respectively).

#### Type of preoperative chemotherapy

All patients received methotextrate/adriamycin/cisplatin (MAP)-based chemotherapy with the exception of a few older adults who received adriamycin/cisplatin (AP) only per standard treatment convention. However, a subset of patients received variant regimens of MAP (mainly MAP/IE (ifosfomide/etoposide)). We found that treatment with a more 'aggressive' regimen was entirely confounded by and highly correlated with whether the patient presented with metastases at diagnosis (Fisher's *P *< 0.001) and did not confer any prognostic value for outcome when adjusted for metastasis at diagnosis. Therefore, there was no need to additionally control the prognostic value of miRNA profile for this covariate.

**Figure 4:**
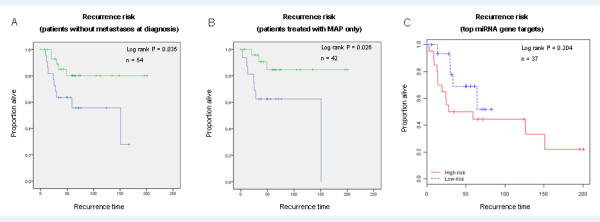
**Recurrence risk prediction in relevant homogeneous patient subsets and using miRNA gene targets**. **(A) **Kaplan-Meier recurrence analysis with the five miRNA profile in the non-metastatic (only) subset of the cohort. **(B) **Kaplan-Meier recurrence analysis with the five miRNA profile in the subset of patients that received MAP (only). **(C) **Kaplan-Meier recurrence analysis using a subset of gene targets of prognostic miRNAs.

To further illustrate the independent prognostic value of the profile we performed Kaplan-Meier analyses restricted to two separate homogeneous subsets of patients, who had non-metastatic disease at diagnosis or who received only MAP chemotherapy. We found that the miRNA profile still retained impressive prognostic power in these homogeneous cohorts, again discriminating between a high and low risk group (median RFS 151 months versus not reached, log rank *P = *0.035; and median RFS 151 months versus not reached, log rank *P *= 0.026; Figure 4A and 4B, respectively).

We reasoned that miRNA risk assessment and chemoresponse may be synergistically prognostic. When chemoresponse was incorporated with the 22 miRNA and 5 miRNA profiles into multivariate models, risk discrimination improved (median RFS: 59 months versus not-yet-reached; HR = 4.96, 95% CI: 1.830 to 13.446, Figure 2B; and median RFS: 59 months versus not-yet-reached; HR = 3.91, 95% CI: 1.533 to 9.956, Figure 2E). We then created a categorical variable taking three possible values: 'poor,' 'intermediate' and 'good.' Patients were classified as 'poor' if they had an unfavorable chemotherapy response and a high-risk miRNA profile. 'Intermediate' patients had either a high predicted risk of recurrence, or an unfavorable chemotherapy response, but not both. Finally, 'good' patients had both a low predicted risk of recurrence, and a favorable chemotherapy response. Kaplan-Meier analysis with this new categorical variable demonstrated a strikingly poor prognosis for patients in the 'poor' category, and significantly better prognoses for both the 'intermediate' and 'good' patients (log rank *P *< 0.001, Figure 2C, F).

The combined power of miRNA expression and chemotherapy response as a clinical covariate was also evident in analyzing OS. miRNA expression levels, taken alone, could not generate statistically significant survival prediction models (HR = 1.65, log rank *P *= 0.365; Figure S1A in Additional file 8). Although this analysis was possibly limited by a small number of deaths, chemotherapy response was predictive of survival (Figure S1B in Additional file 8). However, a combined model using supervised principal components regression identified miR-495 (one of the five miRNAs from the prognostic profile) as significantly adding prognostic power to a model using chemoresponse alone. The combined model including chemoresponse and miR-495 expression showed a very strong discriminatory capacity, despite the limited number deaths in our cohort, (median OS: 82 months versus not-yet-reached, HR = 8.26, 95% CI: 1.820 to 37.435; log rank *P *< 0.001; permutation *P *= 0.11; Figure S1C in Additional file 8). Further refinement of a model for OS would require a future study with a larger sample size.

### Independent external dataset supports the prognostic value of candidate miRNAs for recurrence

Jones and colleagues recently published an independent, publicly available miRNA dataset [7]. Using their dataset, they studied miRNAs relevant to chemoresponse, investigated their biological role, and provided analysis of metastatic outcome based on a more limited sample size (*n *= 29, 10 recurrences). There were substantial differences between this dataset and ours, namely its source (frozen tissue specimens), array platform (Agilent), smaller sample size and event number, and the fact that metastasis was reported as a binary, not time-censored, outcome. However, we performed Cox regression analysis on marker miRNAs included in our 22-miRNA profile, of which only 18 were present on the Agilent array. Of those 18, we found 8 associated with recurrence in the independent dataset with a significant *P*-value (*P *< 0.05) or a trend to significance (*P *< 0.1). Given the sample size and other limitations of this comparison, this finding is significant as confirmed by a simulation analysis testing 100 random lists of 18 miRNAs from the independent dataset that found that only 4 out of 100 contained 8 of the 18 significant miRNAs at the same statistical level (permutation *P *= 0.04), demonstrating that the significance level of our miRNAs in the independent dataset is very unlikely due to chance. We attempted to further use the limited number of deaths in that dataset (*n *= 7), which were reported as a time-censored outcome, and despite the challenge of such a small event number, we were able to generate several models using our prognostic genes showing strong discriminatory capacity for survival, although their *P*-values did not reach nominal significance due to very limited power (Figure 3).

### Chromosomal clustering and target gene regulatory activity analysis of prognostic miRNAs

Interestingly, we observed that a majority of the top miRNA prognostic markers (four from the 5 miRNA profile and fifteen from the 22 miRNA profile) were located at chromosome 14q32. This locus has been associated with Paget's bone disease [36], which is a known strong risk factor for osteosarcoma. At least 10 miRNAs potentially involved in osteosarcoma at 14q32 have been reported [10,15]. However, this locus has not previously been associated with clinical outcome.

Previous work has shown that additional insights into the role of miRNAs can be gained by examining their regulatory activity in terms of effects on target mRNAs [31,37]. Therefore, in a separate analysis of the whole genome DASL data, we explored the association of prespecified gene sets of miRNA targets with recurrence and survival using complementary methods. Using the miRanda algorithm to obtain target gene sets and an established gene set analysis method [31,38], we found a number of miRNA-regulated gene sets demonstrating association with recurrence (*P *< 0.05; Table 3). We also conducted this analysis using the regulatory effects scoring method [31] and identified several miRNAs with significantly different regulatory activity associated with recurrence and survival endpoints (Table 3; *P *< 0.05, FDR < 0.1). Strikingly, among the significant gene sets, some were regulated by miR-411*, miR-379*, miR-539, miR-616*, miR-493*, miR-323-3p and miR-382, which were prognostic of recurrence when their expression levels were assessed. This finding suggests that not only miRNA expression levels but also their target genes (collectively) are associated with outcome by way of deregulation.

**Table 3:** Differential regulatory activity of prognostic miRNAs on the 14q32 locus

Differentially activated miRNA	Gene target prediction	Activity/enrichment assessment	*P*
hsa-miR-758	TargetScan	RE score	0.031
hsa-miR-299	TargetScan	RE score	0.034
hsa-miR-299-3p	TargetScan	RE score	0.034
hsa-miR-493	Pita	RE score	0.022
hsa-miR-323-5p	Pita	RE score	0.025
hsa-miR-411*	miRanda	GSA	0.005
hsa-miR-379*	miRanda	GSA	0.020
hsa-miR-139-5p	miRanda	GSA	0.005
hsa-miR-539	miRanda	GSA	0.047
hsa-miR-616*	miRanda	GSA	0.010
hsa-miR-493*	miRanda	GSA	0.025
hsa-miR-323-3p	miRanda	GSA	0.010
hsa-miR-382	miRanda	GSA	0.040

Using the regulatory effects scoring method with three different miRNA target prediction algorithms and miRanda-based GSA analysis, differentially activated miRNAs associated with recurrence were identified. Listed are those differentially activated miRNAs at 14q32 whose expression levels are associated with recurrence (*P *< 0.05). GSA, Gene Set Expression comparison Analyses; RE, Regulatory Effect

We explored several aspects of the association of the miRNA target genes with outcome. Thirty of the genes targeted by these miRNAs were significantly differentially expressed between the high and low risk groups as defined by the miRNA expression profile (*t*-test *P *< 0.05; Table S4 in Additional file 9). Furthermore, we performed unsupervised hierarchical clustering of the tumor diagnostic biopsy specimens using the expression levels of the gene targets of the prognostic miRNAs. We observed two main clusters, each of them including preferentially high-risk or low-risk samples as defined in the prognostic analysis using the 22 miRNA and 5 miRNA profiles' expression levels (Fisher's exact test *P*-value 0.005 and 0.003, respectively). We also performed supervised recurrence risk prediction analysis and identified a profile consisting of a subset of mRNAs (miRNA gene targets) that showed a strong trend in distinguishing good and poor prognosis tumors, although not as strongly as the miRNA levels themselves - probably as a result of a smaller sample size in this analysis (*n *= 37, median RFS 28 months versus not-yet-reached, log rank *P *= 0.260; Figure 4C). Interestingly, *PDE4PIP*, the top predictor gene in this prognostic analysis, appeared to be targeted by multiple prognostic miRNAs, potentially strengthening the specificity of a biological hypothesis. These findings, taken together, suggest that at least some of the 14q32 prognostic miRNAs together with several of their target genes may also be elements of a deregulated circuit with biological significance in osteosarcoma progression and outcome. Further biological studies are needed, which are beyond the scope of this prognostic study, to further validate these interesting observations and elucidate the full extent of biological significance of these miRNAs in osteosarcoma.

### Chemotherapy response prediction in pretreatment biopsies and expression changes in post-chemotherapy resection specimens

We analyzed chemoresponse as an ordinal binary variable ('optimal' versus 'suboptimal' response defined as the degree of necrosis at the time of definitive resection, assessed by an expert pathologist) using OLR (Figure S2 in Additional file 10). We took the approach of averaging the predictions from the top univariate OLR models, to reduce the chance of serious over-fitting (average univariate prediction; AP). We discovered a range of miRNA signatures (five to ten miRNAs) that predict for optimal chemoresponse with approximately 75% accuracy (averaged over multiple random training/test set splits of different sizes; Figure S3A in Additional file 11, Figure S4A in Additional file 12, and Figure S5 in Additional file 13). The miRNAs in the chemoresponse profile were entirely non-overlapping with the miRNAs in the recurrence/survival profile, underscoring the notion that mechanisms of resistance to conventional chemotherapy may be distinct from mechanisms determining overall outcome. There were 27 miRNAs significant at the 0.05 level and the concordance values of the five to ten top univariate models ranged between 0.67 and 0.76 (Table S5 in Additional file 14). The stability of the predictor miRNA list was assessed in multiple random subsets of the dataset as described in the methods section [39]. We then attempted multivariate ordinal logistic modeling. This analysis was limited by sample size, but we found that a two-miRNA multivariate model performed almost as well as the optimal averaged univariate models (Figure S3B in Additional file 11). No improvement in predictive accuracy was obtained with mRNA models or combined miRNA and mRNA models.

We further performed an exploratory miRNA expression analysis of 26 paired pre- and post-chemotherapy specimens and we observed many expression changes following exposure to chemotherapy (70 miRNAs differentially expressed), which were non-overlapping with the predictive or prognostic profiles described above. This exploratory analysis is described in the Additional file 15 and Table S6 in Additional file 16 and will require further validation in future studies.

## Discussion

Combined modality treatment in osteosarcoma has led to survival gains and fewer amputations, but outcomes have remained unchanged for over 20 years [2,40]. Adoption of novel therapies is complicated by the lack of reliable means to prognostically stratify patients. Even though it is a useful surrogate, pathologically assessed chemotherapy-induced tumor necrosis assessed at the time of definitive resection, the only accepted prognostic variable, is imperfectly correlated with distant outcome especially for sub-optimally responsive patients [2,41]. Genome-wide studies have provided valuable data on chemoresponse [5-7,42,43] but there have been no studies examining miRNA and mRNA expression profiles using continuous time-censored recurrence and survival as endpoints. Given the rarity of well-annotated frozen tissue repositories, we sought to develop clinical outcome predictors using FFPE tissue. Our successful attempt implies clinical applicability, and establishes FFPE tissue as an appropriate substrate for such studies in osteosarcoma, particularly for miRNA profiling.

We found a strong relationship between miRNA expression profiles and RFS, the first such observation in this disease. Using established methods [30], we developed several models predictive of recurrence independent of chemotherapy response, although future development would naturally focus on the smaller, simpler models (for example, the five miRNA profile; Figure 2H). We also demonstrated that miRNA models are prognostic independent of potential confounding by known prognostic factors, such as chemoresponse, tumor location, presence of metastasis at presentation or chemotherapeutic regimen variation (although the latter has not been definitively shown to be prognostic in osteosarcoma to this date). Interestingly, risk prediction improved when chemotherapy response and miRNA risk profiles were combined, suggesting that chemosensitivity and miRNA profiles capture non-redundant prognostic information. Indeed, many patients in our cohort who did not respond optimally to chemotherapy either had no recurrence, or had recurrent disease after a long remission. These findings are highly relevant to clinicians wishing to provide robust prognostic information and prioritize patients for different or novel treatment approaches. For example, the largest ongoing randomized study in osteosarcoma (AOST0331, targeting 1,400 patients worldwide) is studying the modification of chemotherapy postoperatively, in the case of suboptimal response to preoperative chemotherapy. Thus the impetus to incorporate powerful marker profiles, non-overlapping with chemotherapy response, for improved patient risk stratification is increasing. The prognostic association between miRNA profiles and OS was weaker, however the power of this secondary analysis was limited by a smaller number of events (deaths) and we still detected potential prognostic synergy between miRNAs and chemoresponse with respect to OS. A larger future study will allow a more definitive prognostic analysis with respect to OS to be performed.

In addition to a stringent internal cross-validation, we achieved external validation using the only other public miRNA dataset that recently reported outcome data [7]. Although widespread differences between the two studies were challenging (FFPE versus frozen tissue, DASL versus Agilent array platform, continuous time-censored versus binary recurrence outcome, and few events), we were nonetheless able to independently validate the prognostic value of a large subset of miRNAs from our predictive models and were able to develop signatures, using these miRNAs (preselected from our discovery set) with impressive survival distinction, using death as an endpoint. Future studies with larger sample sizes and standardized platforms will take the next step to assess the wider reproducibility and performance of a fully specified model. However, these studies will require a long time to assemble multiple adequately sized tumor cohorts in such a rare disease. Thus, our data provide strong and necessary initial evidence supporting the wider reproducibility of prognostic miRNA profiles in osteosarcoma.

Some of our profile miRNAs have been previously reported [10,15] but not in association with osteosarcoma clinical outcome. These were predominantly located at 14q32 in the genome - a locus associated with osteosarcoma and Paget's disease (a known risk factor for osteosarcoma) - strengthening the biological plausibility of our results. It has been suggested that rearrangements of chromosome 14 may play a role in altered miRNA expression in osteosarcoma [10]. Because miRNA target-genesets of some of 14q32 prognostic miRNAs were also associated with outcome, potentially indicating miRNA activation, our data suggest that this genomic locus may have a significant role in osteosarcoma progression and outcome. Although reports in other tumor systems have suggested a proliferative and pro-invasive role [44], further studies are needed to characterize the precise mechanism by which some of these miRNAs may modulate outcome.

We also investigated the relevance of miRNAs to chemotherapy response and identified novel miRNA signatures predictive of chemosensitivity. These signatures are largely non-overlapping with overall recurrence and survival profiles, supporting the notion that chemoresponse and tumor progression and metastasis may be regulated by non-overlapping molecular networks. We also performed a 'dynamic' analysis, revealing miRNA expression changes following chemotherapy in 'resistant' samples (those with viable tumor at the time of resection). Although this analysis is exploratory and requires further validation in a larger study with additional controls, it is interesting to note that some of the miRNAs identified in this dynamic analysis - for instance miR-15b, and miR-132 - have also previously been reported in relation to chemoresponse [7,11], which is consistent with our results.

mRNA profiles were weaker and did not show additive value. This was possibly due to a smaller sample size (fewer samples run on whole genome expression assays than miRNA assays) and/or unique susceptibility to mRNA damage in FFPE osteosarcoma tissues (compared to miRNA stability) related to the fixation or decalcification process, which might have blunted the biologic signal. Nonetheless, miRNA and mRNA prognostic synergy should be explored more efficiently in future larger studies. Another possible limitation of our study is the inclusion of adult together with pediatric cases. However, only two patients were older than 35 years, making it very unlikely that their potential biologically unique nature affected our results.

## Conclusions

We present the largest archival osteosarcoma profiling study to date. We discovered prognostic miRNA signatures with high discriminatory capacity, independent of and potentially synergistic with traditional chemoresponse assessment, and provided new insights into the molecular networks associated with response and exposure to chemotherapy. Many outcome-related miRNAs display regulatory activity changes related to outcome and share a common genomic locus at 14q32, which has been previously implicated in osteosarcoma. These findings set the stage for studying a sequential prognostic and predictive approach, whereby patients are stratified early based on miRNA profiles at the time of diagnosis, and prognosis is then refined by subsequent pathologic assessment of chemoresponse, potentially with additional contribution from dynamic patterns of miRNA expression in resistant tumors. Finally, our work serves as a model for FFPE systems-based translational and clinically applicable genomic research in rare malignancies with limited tissue availability.

## Abbreviations

AP: average univariate prediction; CI: confidence interval; DASL: cDNA-mediated annealing, selection, extension and ligation; FDR: false discovery rate; FFPE: formalin-fixed, paraffin-embedded; GSA: gene set expression comparison analyses; HR: hazard ratio; miRNA: microRNAs; OLR: ordinal logistic regression; OS: overall survival; RFS: recurrence-free survival.

## Competing interests

JBF and CA are employees of Illumina, Inc. The study used the miRNA microarray product developed at Illumina. The remaining authors declare that they have no competing interests.

## Authors' contributions

ADK and DS conceived the study. ADK, BHK, KEH, EH, AC, NF, JBF, CA and HS performed experiments and analysis. KAJ, JG, KK and ARPA provided specimens and clinical data. ADK, KEH, KAJ, JG, KK, ARPA, MCG, JQ and DS wrote the manuscript. All authors read and approved the final manuscript.

## Supplementary Material

Additional file 1 **Table S1: Detailed clinical characteristics of cohort**. Chemosensitivity-based prognosis in this table is based on chemotherapy-induced tumor necrosis of higher than 80%. Additional relevant clinical characteristics for the cohort are also provided. An asterisk indicates that a paired, post-chemotherapy specimen also exists for this patient. Click here for file

Additional file 2 **Supplementary methods**. Additional details of the methodology are presented. Click here for file

Additional file 3 **Script S1. Regulatory Effects Scoring Script**. The R script used to run the microRNA regulatory effects scoring algorithm described in the methods section. Click here for file

Additional file 4 **Script S2. Average Chemoresponse Prediction Script**. The R script used to run chemotherapy response prediction analysis using the average univariate prediction method described in the methods. Click here for file

Additional file 5 **Script S3. Multivariate Chemoresponse Prediction Script**. The R script used to run chemotherapy response prediction analysis using the multivariate modeling method described in the methods. Click here for file

Additional file 6 **Table S2. mRNAs associated with recurrence**. mRNAs significantly associated with recurrence at the 0.05 level are shown along with FDR, a *P*-value based on random permutations of the data, and a univariate HR. Click here for file

Additional file 7 **Table S3. mRNAs associated with survival**. mRNAs significantly associated with survival at the 0.05 level are shown along with FDR, a *P*-value based on random permutations of the data, and a univariate HR. Click here for file

Additional file 8 **Figure S1. Survival risk prediction of five miRNA profile and chemoresponse**. **(A) **Kaplan-Meier analysis of survival for the five miRNA profile only. **(B) **Kaplan-Meier analysis of survival for chemoresponse only. **(C) **Kaplan-Meier analysis of survival based on miR-495 expression (the miRNA with the strongest parametric *P*-value) combined with chemoresponse as a clinical covariate. Click here for file

Additional file 9 **Table S4. mRNAs differentially expressed across the good and poor prognostic groups**. mRNAs significantly differentially expressed across the good and poor prognostic groups at the 0.05 level are shown along with FDR, a *P*-value based on random permutations of the data, and fold change. Click here for file

Additional file 10 **Figure S2. Comparison of chemosensitivity definition metrics**. These Kaplan-Meier plots demonstrate that the clinically accepted prognostic cutoff for chemotherapy-induced tumor necrosis of 90% performed no better than a cutoff of 80% in predicting risk for recurrent disease for our cohort. Click here for file

Additional file 11 **Figure S3. Predictive modeling of chemotherapy response with miRNA data**. Mean prediction accuracies across 500 iterations of randomly selected training and test sets using (A) the AP method with miRNA data, and (B) the multivariate modeling prediction method using miRNA data. Click here for file

Additional file 12 **Figure S4. Predictive modeling of chemotherapy response**. Mean prediction accuracies across 500 iterations of randomly selected training and test sets using (A) the AP method with mRNA data, and (B) the multivariate modeling prediction method using mRNA data. Click here for file

Additional file 13 **Figure S5. Distribution of accuracies for chemosensitivity prediction**. The example shown is for the AP method using five miRNAs. The distribution of predictive accuracies is shown for (A) 500 iterations of 95/05 (percent of cohort used in training set/percent of cohort used in test set) random splits, and (B) 500 iterations of 90/10 random splits. Click here for file

Additional file 14 **Table S5. miRNAs and mRNAs associated with chemosensitivity**. miRNAs and mRNAs univariately significant at the 0.05 level are shown along with regression statistics. The stability of these top-ranking associated features is reported as a fraction of random subsets of the data in which the highly ranked feature remains highly ranked. Click here for file

Additional file 15 **Supplementary results**. Additional results of the study are presented. Click here for file

Additional file 16 **Table S6. miRNA expression profile changes following chemotherapy**. These miRNAs were found to be significantly differentially expressed at the 0.001 level between paired, pre- and post-chemotherapy tumor samples. Click here for file
