# Supplementary material for: MicroRNA paraffin-based studies in osteosarcoma reveal reproducible independent prognostic profiles at 14q32
Source: Genome Med. 2013 Jan 22;5(1):2. doi: 10.1186/gm406 (PMC3706900; doi:10.1186/gm406)
Supplement: Additional file 2 — Supplementary methods. Additional details of the methodology are presented. [file gm406-S2.DOC]

**Supplementary Methods**

***Quality assessment details***

Dataset quality control metrics included number of probes significantly detected (P<0.01), average signal intensity, 95^th^ percentile signal intensity, and housekeeping gene signal intensity. The data are as follows for the miRNA samples:

Average mean signal intensity: 2162 (1153-3096)

Average 95^th^ percentile intensity: 12263 (4839-18804)

Average probes significantly detected: 548 (354-710)

The data below describes the mRNA samples:

Average mean signal intensity: 1756 (108-4800)

Average 95^th^ percentile intensity: 9488 (43-20285)

Average probes significantly detected: 5989 (828-11861)

Average housekeeping genes intensity: 23041 (3540-35082)

The effect of duration of storage on miRNA and mRNA profiles was assessed via correlation analysis of the samples’ storage age with various array quality metrics.

mRNA assays displayed no significant correlation between storage time and mean intensity (Pearson correlation coefficient = -0.165, p-value non-significant), housekeeping gene signal intensity (Pearson correlation coefficient = -0.050), or intensity at the 95^th^ percentile expression (Pearson correlation coefficient = -0.192, p-value non-significant). There was also no correlation with mRNAs detected at p-value 0.01 or 0.05 (Pearson correlation coefficients = -0.097 and -0.081, respectively).

miRNA assays also showed no significant correlation between mean intensity (Pearson correlation coefficient = 0.065) or intensity at the 5^th^, 25^th^, 75^th^, and 95^th^ expression percentiles (Pearson correlation coefficients = -0.130, -0.040, 0.170, and -0.014, respectively, p-values non-significant) and specimen storage time. The number of miRNAs detected at p value 0.01 or 0.05 showed only minor correlations with the specimens’ storage age (Pearson correlation coefficients = -0.315 and -0.304, respectively). These results suggest that there may be only a minor effect of storage time on a small subset of very lowly expressed miRNAs at a barely detectable level (that could be lost over time). This minor attrition would have only a minimal or negligible bearing on our study findings. Indeed, there was not difference in the distribution of high and low risk prognostic groups between older and more recently stored specimens, and there was not difference in average storage time between specimens in the high and low risk prognostic groups.

***Definition of a chemotherapy response endpoint***

In the literature, osteosarcoma patients are considered to have a favorable prognostic chemotherapy response if tumor necrosis following therapy is 90% or higher (1). Because of the lack of granularity with respect to this endpoint and in order to account for inter-observer variability, we initially split our cohort into five discrete ordered categories corresponding to 0-20, 21-40, 41-60, 61-80, and 81-100 percent necrosis. However, conducting the predictive modeling analysis described in the results using 5 categories proved to require more statistical power than our cohort could provide. Thus, we collapsed the 5 categories into 2 ordinal groups corresponding to 0-80 and 81-100 percent necrosis (unfavorable response and favorable response respectively). A univariate association analysis with the chemotherapy response endpoint demonstrated that the same features were selected for predictive modeling whether we used 5 groups or 2, however, for generating predictions our cohort could only accommodate 2.

To show that an 80% necrosis cutoff is a valid prognostic indicator in our cohort, we performed a Kaplan-Meier analysis of recurrence using both 80% necrosis and 90% necrosis cutoffs to distinguish between high and low risk groups (Additional File 10, Figure S2). In both cases, the high risk group defined by unfavorable chemotherapy response was significantly more likely to have recurrent disease. Furthermore, the median times until recurrence for the high risk groups were very close in the two analyses (in both analyses the median time had not yet been reached for the low risk group). Therefore, in our study, we have evidence that the 80% necrosis cutoff performs as well as 90% necrosis cutoff in assessing risk for recurrence. 80% necrosis was the cutoff used to define chemotherapy response in this part of our study.

***Assessment of chemosensitivity predictor stability***

We tested the stability of predictor miRNAs by generating c-value rankings of the potential chemosensitivity predictors based on each of 100 random subsets of 90% of the biopsy samples, and we computed stability indices as described by Kuncheva (2). In brief, the stability index, which takes possible values between -1 and 1, is a measurement of the cardinality of the intersection of feature lists of equal length. Perfectly overlapping lists will have a stability index of 1, partially overlapping lists due to chance will have a stability index of 0, and entirely non-overlapping lists will have a stability index of -1. In our study, this exercise demonstrated that the top ranked potential predictor miRNAs were consistently very highly ranked across random subsets of the data (Additional File 14, Table S5). Therefore, our results are unlikely to be biased by the method of feature selection.

***Feature selection for multivariate chemosensitivity prediction***

A form of forward feature selection was used in the MP method for chemosensitivity prediction. For all 500 iterations of randomly selected training and test sets an OLR model was trained on the expression of each miRNA for the training sets. The models were ranked by c-value and the miRNA corresponding to the model with the highest c-value was selected as the first independent variable. Then OLR models were trained on the expression of the first independent variable *in addition to* all remaining miRNAs. At this point, the 2 miRNA model with the highest c-value is selected. This process was repeated until a model was built with the desired number of independent variables.

**References**

1. Mintz MB, Sowers R, Brown KM, Hilmer SC, Mazza B, Huvos AG, et al. An expression signature classifies chemotherapy-resistant pediatric osteosarcoma. Cancer Res 2005;65(5):1748-54.

2. Kuncheva L. A stability index for feature selection. In: The 25th IASTED International Multi-Conference: Artificial Intelligence and Applications; 2007; 2007. p. 390-395.
