# Supplementary figures and images for: MicroRNA paraffin-based studies in osteosarcoma reveal reproducible independent prognostic profiles at 14q32
Source: Genome Med. 2013 Jan 22;5(1):2. doi: 10.1186/gm406 (PMC3706900; doi:10.1186/gm406)

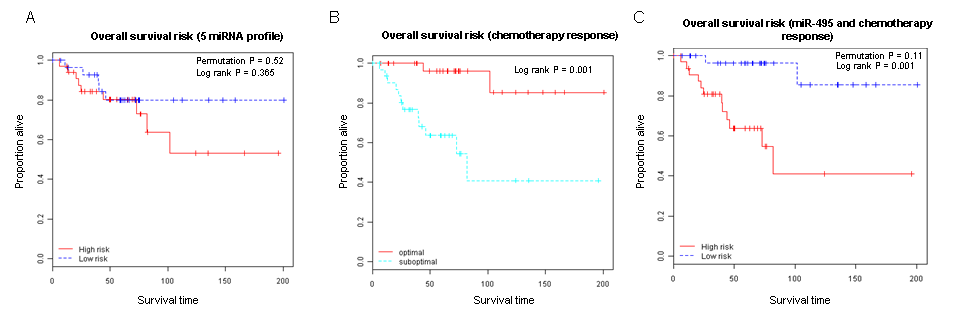

Supplement: Additional file 8 — Figure S1. Survival risk prediction of five miRNA profile and chemoresponse. (A) Kaplan-Meier analysis of survival for the five miRNA profile only. (B) Kaplan-Meier analysis of survival for chemoresponse only. (C) Kaplan-Meier analysis of survival based on miR-495 expression (the miRNA with the strongest parametric P-value) combined with chemoresponse as a clinical covariate. [file gm406-S8.TIFF]

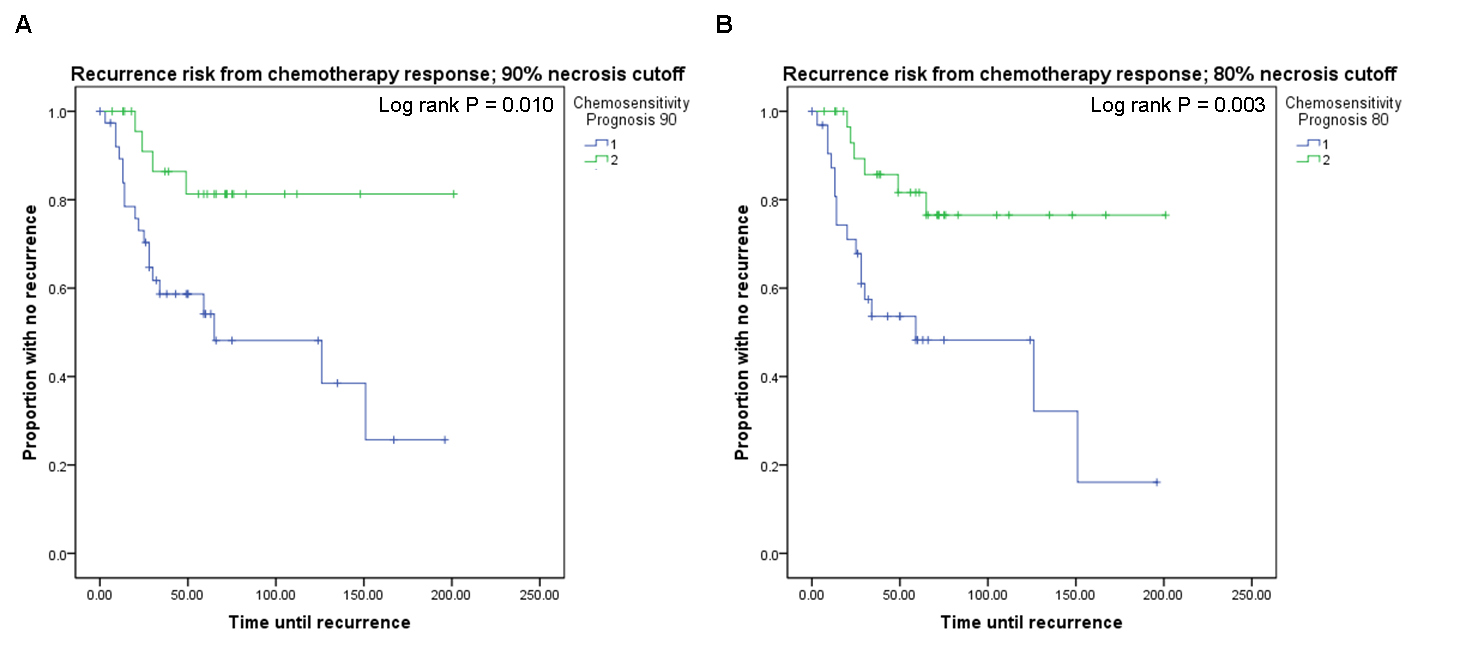

Supplement: Additional file 10 — Figure S2. Comparison of chemosensitivity definition metrics. These Kaplan-Meier plots demonstrate that the clinically accepted prognostic cutoff for chemotherapy-induced tumor necrosis of 90% performed no better than a cutoff of 80% in predicting risk for recurrent disease for our cohort. [file gm406-S10.TIFF]

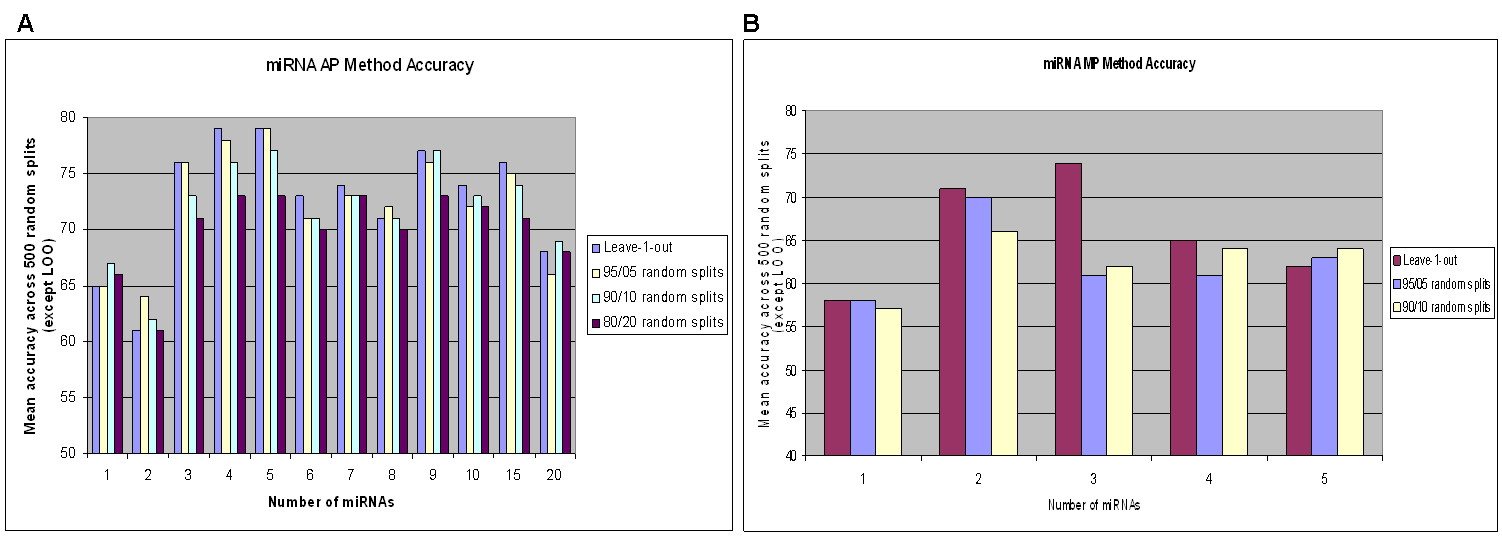

Supplement: Additional file 11 — Figure S3. Predictive modeling of chemotherapy response with miRNA data. Mean prediction accuracies across 500 iterations of randomly selected training and test sets using (A) the AP method with miRNA data, and (B) the multivariate modeling prediction method using miRNA data. [file gm406-S11.TIFF]

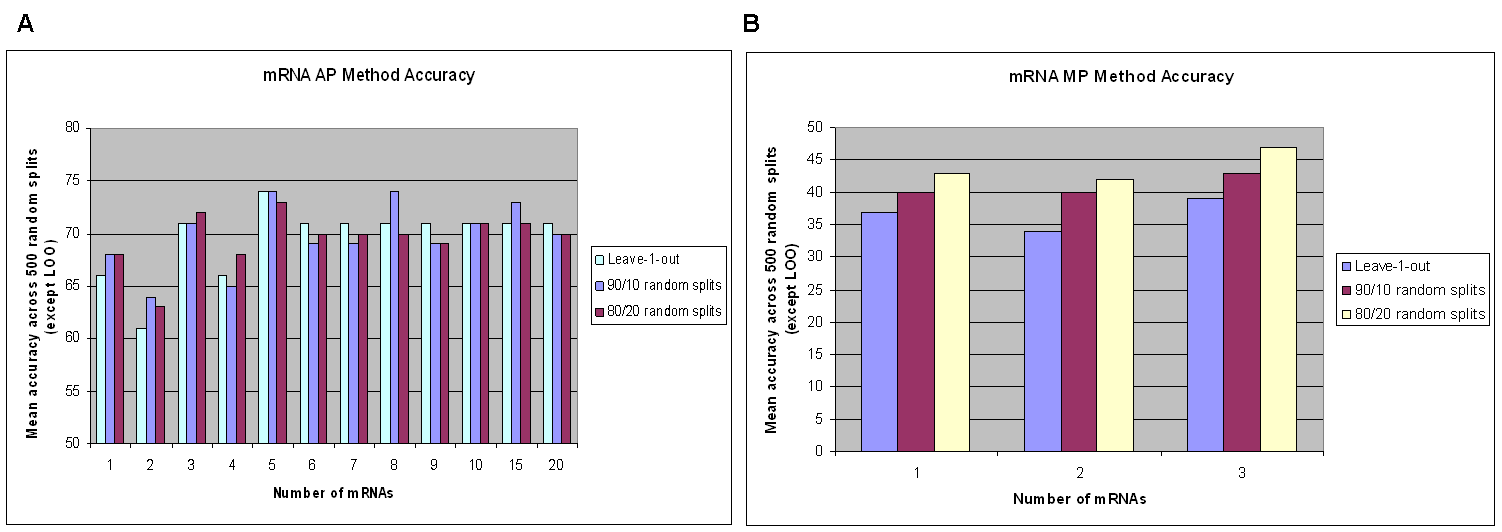

Supplement: Additional file 12 — Figure S4. Predictive modeling of chemotherapy response. Mean prediction accuracies across 500 iterations of randomly selected training and test sets using (A) the AP method with mRNA data, and (B) the multivariate modeling prediction method using mRNA data. [file gm406-S12.TIFF]

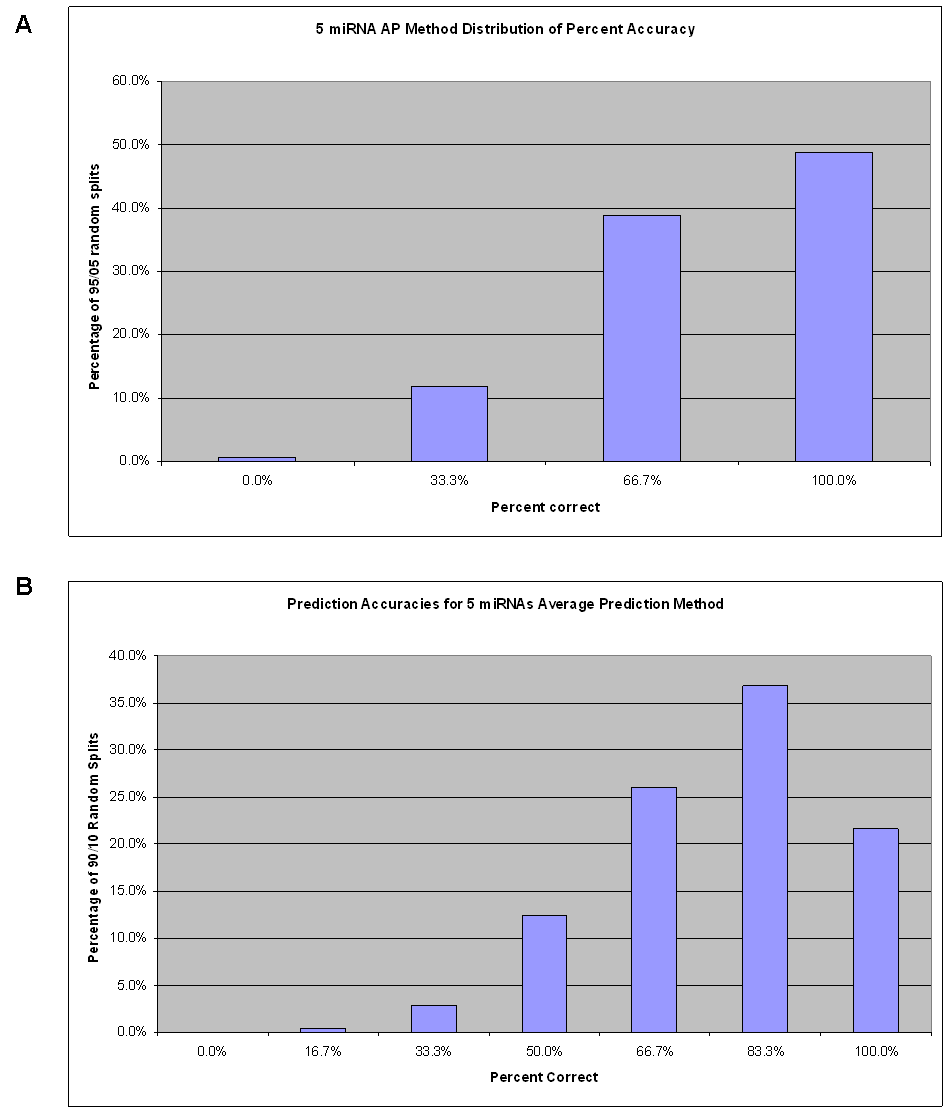

Supplement: Additional file 13 — Figure S5. Distribution of accuracies for chemosensitivity prediction. The example shown is for the AP method using five miRNAs. The distribution of predictive accuracies is shown for (A) 500 iterations of 95/05 (percent of cohort used in training set/percent of cohort used in test set) random splits, and (B) 500 iterations of 90/10 random splits. [file gm406-S13.TIFF]
