# Supplementary material for: MicroRNA paraffin-based studies in osteosarcoma reveal reproducible independent prognostic profiles at 14q32
Source: Genome Med. 2013 Jan 22;5(1):2. doi: 10.1186/gm406 (PMC3706900; doi:10.1186/gm406)
Supplement: Additional file 15 — Supplementary results. Additional results of the study are presented. [file gm406-S15.DOC]

**Supplementary Results**

**Exploratory analysis shows dynamic changes in paired pre and post chemotherapy specimens**

We conducted a paired pre- and post-chemotherapy analysis of the 26 tumor pairs. There were many changes in the miRNA expression profiles of tumors after receiving chemotherapy; we identified 70 miRNAs which were differentially expressed between paired pre- and post-treatment samples (paired t-test p<0.001, FDR <0.02; Additional File 16, Table S6). Of these miRNAs, 20 were up-regulated following therapy, and 50 were down-regulated. There was almost no overlap between the miRNAs showing dynamic expression changes following chemotherapy and the miRNAs presented above in the prognostic profile. mRNA expression analysis did not reveal clear statistical evidence of possible deregulation of target gene sets but this analysis was inconclusive due to the limited amount of WG DASL pairs (5 total) that passed quality controls.
